# Women’s satisfaction with breast reconstruction after mastectomy and a survey on the decision process for type of reconstructive surgery

**DOI:** 10.1007/s00508-025-02526-6

**Published:** 2025-04-02

**Authors:** Ingrid Jelinek, Rupert Koller, Michael Kundi

**Affiliations:** 1 Center for Public Health, Medical University of Vienna, Kinderspitalgasse 15, 1090 Vienna, Austria; 2 Department for Plastic, Aesthetic, and Reconstructive Surgery, Wilhelminenspital, Montleartstraße 37, 1160 Vienna, Austria

**Keywords:** Breast reconstruction, Breast cancer, Autologous tissue, Silicone implants, Oncology, Decision process

## Abstract

**Background:**

Breast reconstruction is an option for women after breast cancer surgery to improve the quality of life. While data about satisfaction after reconstruction are available, little is known about the decision process and about factors shaping this process.

**Methods:**

From 100 selected women, 72 women between 30 and 65 years of age (median 50.3 years, interquartile range 44–57 years) with breast reconstruction conducted in a single center in Vienna (Austria) consented to take part in this study. The role of family, social environment and healthcare providers during decision making, body image, thoughts about hospital stay and potential complications were assessed by a questionnaire. The decision for autologous tissue versus silicone implants was analyzed by structural equation modelling.

**Results:**

Overall, 69% of the women chose autologous tissue either alone or in combination with a silicone prosthesis. Visual appearance was the most important reason (86%) for choosing reconstruction. Thoughts about the stay in hospital and possible complications were important for the mediating role of healthcare providers in deciding on the type of reconstruction. If women had no concerns about complications they made the choice on their own and favoring autologous tissue reconstruction. In contrast, if such concerns existed women tended to seek help from healthcare providers and tended to choose silicone implants.

**Conclusion:**

Counselling of women after breast cancer surgery and during decision making for breast reconstruction should include an esthetic outcome but also possible complications and related length of hospital stay.

**Supplementary Information:**

The online version of this article (10.1007/s00508-025-02526-6) contains supplementary material, which is available to authorized users.

## Introduction

Reconstruction of the amputated breast after breast cancer surgery has great importance for women’s body image. Among women undergoing mastectomy in the USA an estimated 25–50% undergo breast reconstruction (BR) [1–4]. Up to 90% of women with breast cancer reported improvement of emotional, psychological and social well-being after BR and may even contribute to an improved prognosis because it can enhance the woman’s hopes and willingness to survive [5–11].

The BR can be performed using silicone shells or autologous tissue. It can be done immediately after mastectomy (primary reconstruction) or a few weeks or months later (secondary reconstruction). The surgical mode and timing of reconstruction should be discussed with the patient and decided individually [7, 12–17].

Nowadays, silicone implants yield good cosmetic results with low complication rates. Silicone implants are the alternative choice especially for women who are too unstable in health to endure sophisticated tissue transplantation and those who do not possess adequate tissue for reconstruction due to low body mass index (BMI) [18–22].

The use of BR with autologous tissue, the so-called flap surgery, can be performed either as primary or secondary reconstruction and is recommended especially in cases where subsequent radiotherapy is planned. Although silicone implants do not prevent radiotherapy, they can lead to overdosing or underdosing in the target volume of irradiation. Nowadays, three different surgical styles are used: transplanting tissue from the back (latissimus dorsi muscle flap, LD), tissue from the abdomen (transverse rectus abdominis myocutaneous flap, TRAM) or the deep inferior epigastric perforator (DIEP) flap technique, where skin and fatty tissue from the abdomen are used.

According to a comprehensive review [23] comparing satisfaction after implant vs. autologous tissue-based BR in over 8400 patients answering the BREAST‑Q satisfaction with breast scale, measuring a woman’s body image in terms of satisfaction with the breasts (score 0–100 lowest to highest satisfaction) in 14 studies, autologous tissue-based BR provided a benefit for women in terms of satisfaction with the breasts of 8.51 points. This is a substantial difference that speaks in favor of autologous tissue-based BR; however, large heterogeneity was observed and the reasons for the differences between studies are at present not well understood and may derive from differences in surgical techniques, perioperative care or financing of BR treatment. Complication rates were not different between implant vs. autologous tissue-based BR but also this meta-analytical finding was associated with a large heterogeneity. In the Supplementary Table 1, we provide an overview of studies from our scoping review that addressed women’s satisfaction after different types of breast oncoplastic surgery that is in line with the meta-analysis mentioned above.

There are few studies to date dealing with the reasons women with breast cancer have for or against BR. A common denominator of these studies is that women seek restoration of the familiar body image and good esthetic outcome [7, 17, 24–30]. Obstacles are fear of additional surgery, concerns about possible complications, interference with detection of recurrence, time restraints because of family duties but also, although to a lesser degree, systemic factors, such as lack of knowledge about the options and no insurance coverage [3].

Very little is known yet about the conflicting and contrasting thoughts of women in the difficult decision-making process. Patients have to deal with incomplete, insufficient and sometimes dubious information. Too pressing advice can intensify fear and conflicts. Thus, healthcare providers must not only know about the personal conditions, such as life style, social and family conditions but also about the history of illness, complications or comorbidities to give helpful information to the patient [7, 13, 31–33].

A retrospective study demonstrated that patients undergoing mastectomy and BR mainly reported having made their own decision after being informed about BR (66%) rather than reporting having participated in shared decision making (30%) and only 4% of the patients reported the decision was made by the doctor. General and esthetic satisfaction as well as quality of life were highest in informed and shared decision-making patients vs. patients seeking paternalistic providers [34]. Patients who underwent BR were more often informed about it at an early stage, discussed BR with their healthcare providers and were more often involved in the decision process [35]. The frequency and quality of discussing reconstruction influence the choice, regret and satisfaction of the patients [36, 37]. Information about the possibility of BR also helped the patients in decisions about mastectomy. Therefore, the informed decision-making model ensures the best quality of breast cancer treatment decisions [36]. In a Swedish study, lack of patient information and shared decision making negatively influenced immediate BR [38].

The paternalistic model of the patient-health-care provider relationship, with the patient delegating treatment decisions to the health-care provider, is becoming less prevalent in recent decades. It has been considered justified due to the asymmetry of knowledge. In contrast, the informed decision-making model intends to reduce this asymmetry by providing information about the risks and benefits of different treatment options in as clear and unbiased a fashion as possible. This should be done by taking care to invoke not additional fears, thereby enabling the informed patient to voice a treatment preference ultimately leading to shared-decision making [39]. Therefore, inquiring about the decision process needs to address the way information was received by the patient and about its sufficiency to make a decision.

In order to scrutinize the evidence base concerning BR satisfaction (study aim i), we performed a scoping review. To further investigate the decision process before BR (study aim ii), an own survey was conducted.

Hence the study aims were:

i) to get an overview of women’s satisfaction with BR after mastectomy,
ii) to gain more insight into the decision process before BR and to determine the factors responsible for a choice of silicone versus autologous tissue implants.

While the latter aim may be best assessed in prospective studies, a thorough consideration led to the decision to not interfere with the process while BR was still pending as this could lead to a change of the opinion of the women, and to perform a retrospective study in women that have already undergone BR.

## Material and method

### Scoping review

The scoping review was performed by searching PubMed and Scopus using the search terms “breast reconstruction”, “breast cancer”, “mastectomy”, and “satisfaction”, restricting the search to publications after 2010, original research and to the English language. The search revealed 207 articles. After exclusion of duplicates, we screened 179 articles by title and abstract and excluded 74 articles because of other outcomes than satisfaction, 59 articles were excluded due to a focus on other therapeutic components (e.g. irradiation, timing of surgery) and 8 articles were excluded after checking the report because of insufficient data.

### Survey in women after breast reconstruction

#### Patient selection

The sample size was determined based on the intended estimation of coefficients for a structural equation model with 210 degrees of freedom. The sample size should be sufficient to discriminate between a moderate model fit with a root mean square error of approximation (RMSEA) of 0.08 and a good fit of RMSEA = 0.05. For this purpose and choosing an alpha error of 0.05 and a power of 80% a sample size of at least *n* = 82 is needed. Assuming a participation rate of 85%, a sample size of *n* = 97 rounded up to *n* = 100 was chosen. Given this sample size, the power to detect an effect size of Cohen’s d = 0.8 at the global alpha level of 5% for the comparison of single variables between types of BR is approximately 85%.

From the files of all women who underwent BR after breast cancer surgery at the department of Plastic, Aesthetic, and Reconstructive Surgery of the Wilhelminenspital, Vienna, Austria, women were selected by simple random sampling. Inclusion criteria were surgery performed between 2005 and 2013, age between 30 and 65 years at the time of surgery and able to fill in a questionnaire in German. The study was approved by the Ethics Committee of the Consortium of Viennese Hospitals.

Selected patients received a letter with information about the study and a self-report questionnaire as well as a prepaid postal envelope for returning the questionnaire. Together with the questionnaire women returned the written consent to use the data by the research group for scientific purposes.

##### Questionnaire

The questionnaire was compiled based on previous studies [40–42] and consisted of four parts. 1) General questions about the reasons for BR, method of reconstruction and expectations concerning the result of reconstruction. 2) Personal feelings about the reconstruction and the role of body image and self-esteem, the role and importance of the opinion of the partner, family and friends, questions concerning thoughts about stay in hospital and possible complications, questions about the influence of different information sources and of expert information and discussion with the healthcare providers involved. 3) Questions about communication with healthcare providers. 4) Sociodemographic data including age, profession, partnership, opinion of the family and questions about the social role of the patient. The face validity of the questions was checked independently by three experts. A pilot version was tested for clarity and comprehensibility in a panel of five women.

The first part consisted of five single choice questions about the main reason and time point of the decision for BR, the method applied, the main reason for choosing this method and the role the partner and family played in this decision.

The second part, about general feelings, consisted of a part with four questions about the role of body image (e.g., How important was your appearance in usual dress for your decision?) with a Likert type answer format with four categories (from “unimportant” to “important”), another part with four questions about the importance of partner, family, and friends in the process of decision making considering the time before surgery (e.g., How strong did your partner’s opinion influence your decision? or how strong have family, relatives and friends influenced your decision about breast reconstruction?), three questions about participation of the partner in the decision process (Did your partner actively participate in the decision process? How important were the thoughts of the partner for your decision? Has the attitude of your partner influenced your decision?), two questions about thoughts circling around complications and lengthy hospital stay, about the influence of personal opinions of healthcare providers contacted, all with the same answer format with four categories from “not at all” to “very strong influence”.

The third part consisted of five questions about the source and importance of information about BR. One single choice question about the primary source of information about BR, one single choice question about the primary source of information among health-care providers, and which one provided the most comprehensive information, one question about whether or not the women considered the information provided sufficient (four categories: “not sufficient” to “completely satisfactory”) and one question about the importance of the information received by healthcare providers during decision making (three categories “not important”, “important”, “very important”).

The last part consisted besides questions about age, profession at the time of surgery, and partnership, questions about the importance of the woman’s role in the family and fear about losing one’s job (four categories from “not at all” to “very important”), and three questions about the importance of reactions of family members.

##### Statistical methods

Data analysis was performed by SPSS 23.0 (IBM Corp., NY, USA) and Statistica 12 (Cloud Software Group, Inc., Palo Alto, CA, USA). Comparisons of groups differing in method of reconstruction were done by Fisher’s exact probability tests for nominal data, Kruskal-Wallis tests for ordinal data and ANOVA for metric data. In the last case normality was assessed by the Shapiro-Wilk test and homogeneity of variances by Levene’s test. For the purpose of structural modeling, scales were area transformed to obtain a normal distribution of scores. Structural models were fit by using SEPath from Statistica 12. Model coefficients with *p*-values against the hypothesis of a zero effect below 0.1 were kept in the model. For statistical comparison of groups a *p*-value below 0.05 was considered significant.

## Results

Overall, 100 patients were extracted from the files of the department of Plastic, Aesthetic and Reconstructive Surgery of the Wilhelminenspital, Vienna, Austria. Of these patients, 5 were deceased and 95 eligible patients received the postal questionnaire. Of these women, 72 completed the questionnaire and sent it back (response rate 76%). The median age of respondents at the time of surgery was 50.3 years (interquartile range 44–57 years), with a range between 30 and 65 years. Nonresponders did not differ by age (median 50.2 years, range 36–65 years, *p* = 0.881) or method of BR (silicone 39% vs. 36%, *p* = 0.822). Reasons for nonparticipation were moved to unknown address (*n* = 11) and too busy (*n* = 12). Of the women three (4%) had severe complications and needed a second surgery (two with silicone implants and one with autologous tissue). All women were Austrian citizens, 70 women (97%) were married or in a fixed partnership and 65 women (95%) were in an active employment at the time of surgery. The time of the BR was between 1.5 and 10 years before the survey (mean ± SD, 5.6 ± 2.7 years). Contrasting patients with time since surgery in the first quartile (below 3 years) with those above the third quartile (7.7 years) revealed no indication of a relevant influence on the study variables (all *p* > 0.20).

Of the participants 61% chose autologous tissue, with 46% choosing autologous tissue without and 15% with silicone and 39% decided for silicone implants. Among women who chose autologous tissue, 61% voted for surgery with tissue from the abdomen by TRAM or DIEP and 39% chose LD flaps. Primary reconstruction was chosen by 43 women (60%), while 29 women (40%) preferred secondary reconstruction.

The vast majority (86%) mentioned visual appearance as the reason for choosing reconstruction. Medical (24%) and other reasons (19%) were present as the sole or as additional reasons in smaller numbers of women (women could select more than one reason). Concerning the choice of method of reconstruction, nearly a half (47%) gave medical reasons, with 10% mentioning a planned irradiation before reconstruction. Other reasons were length of hospital stay or stress of surgery (21%) and expected visual appearance (26%). The remaining women (6%) gave no specific reason for the choice of method.

Women choosing autologous tissue reconstruction did not differ in age from women choosing silicone (50.6 ± 8.1 years vs. 50.0 ± 9.8 years). There was no significant difference according to the type of surgery in reasons for choice of reconstruction (Table 1).

**Table 1:** Attitudes of women towards different aspects of breast reconstruction and importance of aspects of body image for the decision by choice of silicone or autologous tissue for reconstruction

	Silicone	Autologous tissue	Total	*p*-value
	*n* (%)	*n* (%)	*n* (%)	
*Reasons for choice (more than one answer possible)*				
Medical	5 (18)	12 (27)	17 (24)	0.359
Visual appearance	25 (89)	37 (84)	62 (86)	0.534
Other reasons	5 (18)	9 (20)	14 (19)	0.786
*Hopes about outcome*				
Appearance as before surgery	2 (7)	3 (7)	5 (7)	0.911
Little difference to healthy breast	4 (14)	8 (18)	12 (17)	–
Satisfactory appearance	22 (79)	33 (75)	55 (76)	–
*Time of decision*				
Immediately after diagnosis	3 (11)	12 (27)	15 (21)	0.195
After some time of consideration	12 (43)	13 (30)	25 (35)	–
After consultation with healthcare providers	12 (43)	19 (43)	31 (43)	–
After discussion with partner/family	1 (4)	0 (0)	1 (1)	–
*Role of social environment during decision making*				
Strong influence	1 (4)	1 (2)	2 (3)	0.497
Some influence	3 (11)	2 (5)	5 (7)	–
Hardly an influence	14 (50)	18 (41)	32 (44)	–
No influence	10 (36)	23 (52)	33 (46)	–
*Which family members were most important during decision making*				
Partner	23 (82)	24 (55)	47 (65)	0.022
Children	6 (21)	13 (30)	19 (26)	0.586
Parents	2 (7)	2 (5)	4 (6)	0.640
Others	1 (4)	7 (16)	8 (11)	0.139
*Role of healthcare providers’ personal opinion during decision making*				
Strong influence	4 (14)	8 (19)	12 (17)	0.440
Some influence	15 (54)	23 (53)	38 (53)	–
Hardly an influence	8 (29)	7 (16)	15 (21)	–
No influence	1 (4)	5 (12)	6 (8)	–
*Influence of the longer hospital stay on the decision*				
Very strong influence	6 (21)	4 (9)	10 (14)	0.167
Rather strong influence	15 (54)	21 (48)	36 (50)	–
Not at all	7 (25)	19 (43)	26 (36)	–
*Influence of risk of complications on the decision*				
Very strong influence	1 (4)	0 (0)	1 (1)	0.052
Rather strong influence	5 (18)	1 (2)	6 (8)	–
Hardly an influence	18 (64)	32 (73)	50 (69)	–
Not at all	4 (14)	10 (23)	14 (19)	–
*Influence of information from healthcare providers during decision making*				
Very strong influence	21 (75)	33 (75)	54 (75)	0.977
Rather strong influence	6 (21)	9 (20)	15 (21)	–
Not at all	1 (4)	2 (5)	3 (4)	–
*Importance of natural appearance for the decision*				
Important	18 (64)	30 (68)	48 (67)	0.940
Rather important	7 (25)	10 (23)	17 (24)	–
Rather unimportant	3 (11)	4 (9)	7 (10)	–
Unimportant	0 (0)	0 (0)	0 (0)	–
*Importance of body image for the decision*				
Important	20 (71)	33 (75)	53 (74)	0.970
Rather important	6 (21)	9 (20)	15 (21)	–
Rather unimportant	1 (4)	1 (2)	2 (3)	–
Unimportant	1 (4)	1 (2)	2 (3)	–
*Importance of feeling attractive*				
Important	11 (39)	12 (27)	23 (32)	0.547
Rather important	10 (36)	23 (52)	33 (46)	–
Rather unimportant	6 (21)	7 (16)	13 (18)	–
Unimportant	1 (4)	2 (5)	3 (4)	–
*Intensity of thoughts about others noticing the bodily change*				
Very strong	5 (18)	10 (23)	15 (21)	0.840
Rather strong	11 (39)	14 (32)	25 (35)	–
Hardly strong	9 (32)	13 (30)	22 (31)	–
Not strong	3 (11)	7 (16)	10 (14)	–

About one fifth (21%) of women decided for reconstruction immediately after diagnosis and 79% decided after some time of consideration, after consultation with healthcare providers or the partner. Hopes about outcome of reconstruction were realistic (with 76% overall hoping for an acceptable appearance). Little difference to the healthy breast and appearances as before surgery were hoped for in 17% and 7%, respectively.

Independent of the type of surgery chosen, the social environment had little influence on the decision (only 10% reported strong or some influence). Such influence was especially exerted by the partner, in particular if silicone reconstruction was chosen (for 55% of those choosing autologous tissue vs. 82% of those choosing silicone implants the partner was most important, *p* = 0.022).

While the information received by healthcare providers (and especially by plastic surgeons) was important for almost all women, the personal opinion of these healthcare providers about reconstruction had little impact on the decision.

For some 90% the physical appearance was of importance in considerations about choice of reconstructive surgery. Likewise, body image (important in 94%) and feeling attractive (important in 78%) played a role in decision making and more than half of the women (56%) had intensive thoughts about how they will appear to others (Table 1).

**Fig. 1:**
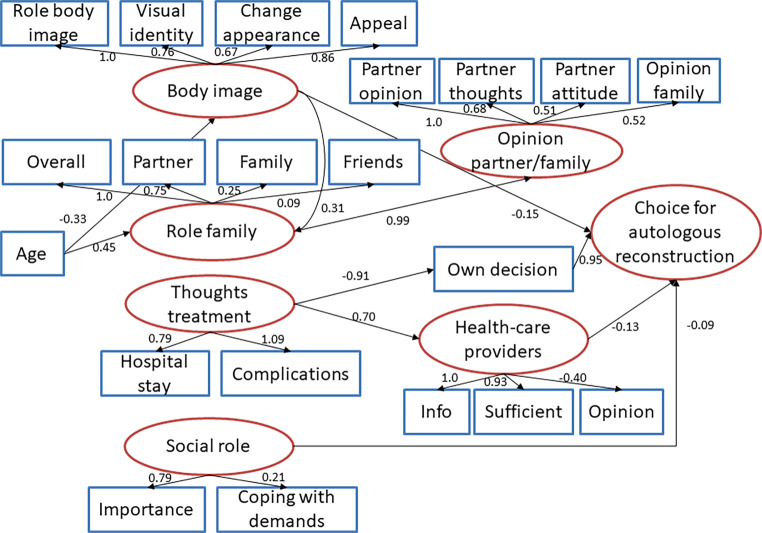
Path diagram of the relationships between the different variables of interest with respect to the decision for autologous tissue reconstruction (against choice of silicone) in women after breast cancer surgery. Standardized coefficients are shown in the diagram. Observed variables are indicated by squares, latent variables by ovals. Paths with *p*-values for the coefficients below 0.1 are kept in the diagram

There were only small differences in essential aspects of decision making in women who chose autologous tissue as compared to those who chose silicone; however, the structural aspects of decision making cannot be extracted from comparison of each component alone. Therefore, structural equation modelling was applied to elucidate the essential pathways that led to the decision for or against autologous tissue. Figure 1 shows the path diagram. It is apparent that there is only one main path of decision making that starts with thoughts about the procedure in the hospital and is concerned with length of stay and possible complications. If these concerns are strong then counselling by healthcare providers is particularly important and tends to lead to favoring silicone reconstruction. On the other hand, if there are no or little concerns about the hospital stay and complications women tended to decide on their own and favor autologous tissue reconstruction. Analysis revealed only two other but weakly influential paths: one starts from the body image and leads to favoring silicone if the women were greatly concerned about attractiveness and the other starts from the social role and its importance and from the perceived difficulty in fulfilling the social demands that, if deemed substantial by the women, also tended to lead to choosing silicone reconstruction.

## Discussion

In our study, 69% of the patients decided to have reconstruction with autologous tissue, either solely or in combination with silicone and 60% of the patients chose primary reconstruction.

Some studies found a tendency of women favoring silicone implants if they intend to very soon go back to normal life and retain their former body image [13, 43]. On the other hand, autologous reconstruction is considered by others to yield a more satisfying body image. Using one’s own tissue may lead to a natural shape of the breast, the reason why many women choose this method in spite of additional surgery and additional scars [44–48]. Choosing the DIEP method, patients expected the best natural appearance and a more natural body feeling than with a silicone implant [47].

In 186 patients with either LD, LD + implant, TRAM or expander/implant, the autologous reconstruction was the preferred method and yielded the highest satisfaction despite more complications and additional scars. For autologous reconstruction, given a thorough selection of patients, a high postoperative satisfaction can be achieved [46].

Few authors distinguish between the modes of reconstruction. In one study patients reconstructed with LD methods showed the lowest rates of complications, compared with free TRAM flaps [49]. Comparing the results after TRAM and DIEP reconstruction, the DIEP group suffered less from complications [50].

For 86% of the patients in our study, restoration of the body image was the most important aspect, irrespective of the type of reconstruction. For 90%, natural appearance was important and 76% wanted at least a satisfactory result. These results are consistent with other studies concerning expectations of women with breast cancer facing BR. Feeling normal is the most important wish that women express before BR [13, 24, 25, 28, 51].

According to our own survey, discussion with health-care providers was important for 94% of all patients and 65% of women in a partnership sought the partner’s opinion. Personal expectations and personal feelings, however, had the greatest influence on decision making for BR. On the other hand, comprehensive information and support by healthcare providers could play a mediating role in choosing the method of reconstruction. As has been emphasized, the decision for immediate reconstruction is correlated with younger age and with the surgeon’s recommendation [52].

After a diagnosis of breast cancer and facing the necessity for mastectomy, a woman has to decide upon whether a BR should be done and which method should be applied. In such a stressful situation clear and balanced information by healthcare providers about the pros and cons are of great help. Surgery at a sexually attributed organ can raise special problems for the patient. As our study showed, for most of the women their individual expectations and hopes are decisive and they circle around maintaining an attractive appearance. Women’s own thoughts have priority; partner’s opinion is often asked for but plays no important role in the end. The opinion of friends and other members of family are relatively unimportant.

Concerns about treatment (length of stay in hospital and potential complications) play an important role for some women during the decision process. Although not statistically significant, women choosing autologous tissue BR had less fear of complications. If the patient has few fears in this respect, she tends to decide on her own and in this case prefers autologous tissue reconstruction. Quite understandably, if she expects complications or a prolonged stay in hospital she seeks advice from healthcare providers, which leads in this case to favor silicone implants. In a previous study it was also found that thoughts about complications are of great importance during decision making [53]. It also has been shown that the decision for the type of reconstruction is correlated with patient participation in decision making and information from healthcare providers [38].

The decision for or against reconstruction, for the mode and timing of surgery is strongly influenced by information from healthcare providers; however, based on this information women decided on their own at least when they considered the procedure as relatively safe. Earlier studies underline the importance of information especially for autologous tissue reconstruction [24, 25, 38, 44, 52, 54, 55]. It has also been emphasized that the mental situation for decisions concerning surgery affords informational support [5, 28, 32, 56–59]; however, technical possibilities and optimal solutions for the women concerned are not always the focus of counselling but preferences and practices of the clinic and the personal opinion and expertise of the surgeons [14, 24, 25, 44, 52, 56, 60].

Although the limited number of patients included in this study prohibits far-reaching conclusions, the strength of this study is its focus on patients of a single department avoiding too much heterogeneity in treatment, information provided to the women and the counselling process. On the other hand, restricting the analysis to women treated at a single center may limit generalizability, although structural relationships can be less affected by the sample chosen; however, the fact that in Austria BR is fully covered by the mandatory general health insurance will make the results less applicable to regions where costs for BR could be a relevant obstacle for the choice. The limited sample size also prohibited conclusions about the tissue chosen for autologous tissue BR. Furthermore, the women in our study were comparatively young and predominantly in a partnership, limiting conclusions about older or single women. This study focused on the decision process from the perspective of the woman, including the views of family members. Our study was retrospective, which could introduce bias as in hindsight the view on the decision process could have been changed by the outcome. Another limitation is the lower participation rate than expected. As nonparticipants did not differ according to type of BR and age, and reason for nonparticipation was likely unrelated to the outcome of BR, it might be assumed that no substantial bias was introduced from the imperfect participation rate.

## Conclusion

Personal expectations determine the decision-making process of women with breast cancer concerning BR. Consultations with healthcare providers are of great importance in particular if there are concerns of the women about complications and length of stay in hospital. Providing detailed information about the options and consideration of the individual’s medical, social and personal situation are important, while the personal opinion of the medical staff is of little help to the women.

## Supplementary Information

Supplementary Table 1: Overview of studies investigating satisfaction with breast reconstructive surgery after mastectomy

## Data Availability

The datasets generated and analyzed during the current study are not publicly available due to restrictions in the informed consent but are available from the corresponding author on reasonable request.
