# Supplementary material for: Women’s satisfaction with breast reconstruction after mastectomy and a survey on the decision process for type of reconstructive surgery
Source: Wien Klin Wochenschr. 2025 Apr 2;137(19-20):619–27. doi: 10.1007/s00508-025-02526-6 (PMC12534258; doi:10.1007/s00508-025-02526-6)
Supplement: Supplementary file 1 — Supplementary Table 1: Overview of studies investigating satisfaction with breast reconstructive surgery after mastectomy [file 508_2025_2526_MOESM1_ESM.docx]

Supplementary Table 1: Overview of studies investigating satisfaction with breast reconstructive surgery after mastectomy

| **Study** | **Region** | **Sample size** | **Sample characteristics (age)** | **Type of surgery** | **Assessment of satisfaction/time after surgery** | **Satisfaction outcome** |
| --- | --- | --- | --- | --- | --- | --- |
| Ashraf, Colakoglu [1] | USA | 465 | 25.5-70.7 y | Autol., autol.+implant, implant/expander | SF-12, 5-Likert scale/ ≥1 y | Corr. between phys./emot. wellbeing and information |
| Augustinho, Neto [2] | Brasil | 90 | 30 - 55 y | Mastectomy/LD/TRAM | Breast Evaluation Questionnaire (BEQ) 55 items | Higher satisf. In LD/TRAM groups |
|  |  |  |  |  |  |  |
| Benditte-Klepetko, Lutgendorff [3] | Austria | 126 | 33-82 y | DIEP, LD, TRAM, Implant | study-specific questionnaire, SF-36/6-77 months | TRAM and DIEP: ‘high’ satisfaction > 80% |
| Caputo, Vigato [4] | Italy | 192 | Mean 53.5 y | Natrelle 410, one stage/ two stage BR | BREAST-Q/4-43 months | High satisf. with outcome score 82/84, no significant differences between groups, medium satisf. with information (score 65/60) |
| Cordeiro, McGuire [5] | USA | 2795 | 18-82 y | TE 91.8%/Implant | 5-point-Likert scale/2 y | 91.1% definitely /somewhat satisfied in general, 97.1 satis. with shape |
| Damen, Timman [6] | Netherlands | 186 | 20-69 y | TE/implant (n=70), LD+/-implant (n=47), DIEP/fTRAM (n=49) | 3-tyer/1-5 y | TE/Implant: 46% LD±implant: 70%  DIEP/fTRAM: 84% |
| Dauplat, Kwiatkowski [7] | France | 423 | 25 – 86 y | Mastectomy (n=122)/IBR (n=301) | QLQ-C30, QLQ-BR23, +motivation quest. + quest. for satisf.  cosmetic outcome/6 – 12 months | Higher scores in IBR group, scores rising from 6 to 12 months |
| de Blacam, Momoh [8] | Ireland | 61 | 28-76 y | Autol., implant, autol+implant | BREAST-Q/≥2 y | Sign. correl. between satisfaction with surgeon/satisfaction with outcome and sat. with reconstr. |
| Dean and Crittenden [9] | Australia | 343 | 24-82 y | Autol. & Implant, | BREAST-Q/1-12 months | Higher satisf. with autol. |
| Di Micco, O'Connell [10] | UK/Italy | 168 | 33-84 y | Wise pattern 87.5%  Other risk reducing techniques | BREAST-Q/13-77 months | 73.5% breast, 75% psychological+physical wellbeing |
| Du, Zhou [11] | China | 63 |  | LD immediate (n=56/delayed (n=7) | Special questionnaire/3 – 28 months | Cosmetic result 58.7% excellent + 30.2% good |
| Fallbjork, Karlsson [12] | Sweden | 126 | 36-79 y | Mastectomy 33%, delayed implant 76% | LAM, EORTC-BR23/ 2-3 y | BR-group higher satisf. in attractiveness/body disclosure  82% self-decision |
| Gschwantler-Kaulich, Schrenk [13] | Austria | 48 | 25-72 y | Comp. of 2 implant types (TILOOP, Protexa) | EORTC QLQ C30, BR23, 4-point-Harris scale 4-yter/2 weeks to 6 months | 87.5% satisf. with TILOOP and less complic., 79% satisf. Protexa |
| Heneghan, Prichard [14] | Ireland | 179 | 27-74 | LD, LD+Impl. TRAM, Impl | EORCT-B23, FACT-B/ 12-70 months | 89.5% satisfied (autol. transpl) |
| Ho, Hartman [15] | Canada/USA/UK | 510 | 21-81 y | TRAM, DIEP, silicone, saline | BREAST-Q | Correlation information-satisfaction with breasts and overall outcome |
| Howes, Watson [16] | Australia | 400 (87 BR) | 20-87 y | Breast conservation/mastectomy with/without BR  BR-group: immediate/mixed/delayed | BREAST-Q/3-114 months | Satisf., psychosocial wellbeing/sexual wellbeing higher scores with BR  BR-group: Imm. 74%  mix 82%, del 76% satisf. |
| Hunsinger, Hivelin [17] | France | 111 | 45->74 y | DIEP | SF-36/5-15 years | Higher scores (48-89) esp.emot. role compared to mast. alone (42-70) and mast.+BR (44-74) |
| Juhl, Christensen [18] | Denmark | 144 | 25.5-73.4 y | Abdom., LD, implant+TD, immediate (different rec. types) | Hopwood’s Body Image Scale, Beck Depression Inventory, Impact of Event Scale, study spec. scale (satisfaction) 7-tyer, 5-Likert Scale/1.1-7.2 y | 83.9% satisfied with decision |
| Kelsall, McCulley [19] | UK | 567 | Mean 54.9/51.8 y | BCS (n=286)/BR (n=281) with DIEP (65), LD (105), implant (111) | Hopwood Body Image Scale + specific questionnaire | Higher scores in BR group concerning body image >> sign. difference |
| Liu, Chiang [20] | China/USA | 74 | 40-57 y | Expander-Implant (EIBR) or DIEP, MS-TRAM (MAFBR) | BREAST-Q | 75->90% satisf. in MAFBR-group without complications |
| Liu, Li [21] | China | 43 | 28 – 59 y | LD+implant (n=29)/LD without implant (n= 14) | Harris method/3-52 months | 65% excellent cosmetic result (LD+implant), 57% excellent c.r. (LD only); over all 83.7% satisfied with cosmetic result |
| Mericli, Szpalski [22] | USA | 47 | Mean 50.3 y | LD immediate+delayed | BREAST-Q/1-11 y | High scores in psychosocial and physical wellbeing (mean 87), mean score 61 in satisf. with breast |
| Momoh, Colakoglu [23] | UK | 346 | <40->60 y | TRAM, DIEP | Modified BREAST-Q/4-144 months | overall satisfaction 81.7% DIEP, 70.2% TRAM  aesthetic satisfaction 72.5/77.2 |
| Nelson, Allen [24] | USA | 3268 | Mean 49.6 y | 2932 Implant (IBR), 336 TRAM, DIEP, SIEAP (ABR) | BREAST-Q/1-8 y | Satisfaction higher in ABR-group, rising with time |
| Qin, Tan [25] | China | 151 | 23-58 | Autologous (n=59)/implant (n=54)/expander-prosthesis (n=38) | QLQ-C30  and QLQ-BR23/12 months | Higher satisf. In expander group, no sign. differences |
| Rindom, Gunnarsson [26] | Denmark | 40 | > 18 y | LD (n=18) /thoracorsal artery perforator flap (TAP, n=22) | QLQ-30, BREAST-Q/12 months | Slightly higher overall outcome with LD (no sign. difference), higher score in physical wellbeing in TAP-group (80.3:78.6) |
| Roje, Roje [27] | Croatia/Austria | 101 | 25-74 y | TRAM, LD, LD+Impl/expander, expander/implant | Modified SF-36 | No differences between BR types, social life significantly improved |
| Santosa, Qi [28] | USA | 1531 | <45 - > 60 y | Implant, autologous | BREAST-Q/2 y | no age effect for physical and psychos. wellbeing in implants, higher scores in older women with autologous BR (scores 70-80) |
| Santosa, Qi [29] | USA | 2013 | Mean 48.1 y (implant),  51.6 y (autol.) | 1490 implant, 523 autologous (DIEP, TRAM, SIEAP) | BREAST-Q/1-4 y | Autologous scores rising with time, higher in satisfaction and psychos. wellbeing (>70) |
| Semmler [30] | Germany | 32 | 30-70 y | 19 DIEP, 13 LD | SF-36, special designed questionnaire 6-tyer/45 months | Higher satisf. with DIEP in shape, higher satisfy. with LD in naturalness |
| Sgarzani, Negosanti [31] | Italia | 63 | 31-74 y | 33 DIEP, 30 exp./Impl | BREAST-Q/> 2 y | DIEP: ‘high’ satisfaction esp. in naturalness (78.7%) and shape (85.7%) |
| Spector, Mayer [32] | USA | 21 | 20-65 y | Implant, TRAM | BREAST-Q, interview/n.s. | Positive feelings about reconstr. and outcome, longer recovery with TRAM |
| Szutowicz-Wydra, Wydra [33] | Poland | 305 (79 BR) | Mean 50.8 y | LD, TRAM, implant | EORTC QLQ C30, BR23/9-12 months | BR-group sign. higher phys.funct. (87 vs. 74), higher emot.funct. and role funct., slightly better body image in BCT-group |
| Tallroth, Velander [34] | Sweden | 73 |  | DIEP, expander | BREAST-Q/30 days | Higher satisfaction with DIEP |
| Temple-Oberle, Ayeni [35] | Canada | 123 | Mean 46.7 y (implant), 51.7 y (alloplastic), 51.9 y (autologous) | Autologous, alloplastic, LD/implant | BRECON-31 | Satisfaction with LD/implant very high (score 91.1), other scores > 80 |
| Wang, Wang [36] | China | 126 | 28-56 y | Silicone implants | 2-tyer/6-12 months | 98% |
| Wattoo, Nayak [37] | UK | 188 | 25-71 y | LD, LD/implant | BREAST-Q/2-12 y | LD/implant sign. higher overall satisf. (score 78.9 vs 66.4) higher outcome satisf. (81.3 vs. 68.7) and higher phys. wellbeing (79.9 vs. 63) |
| Xu, Lei [38] | China | 1554 | Mean 40.7 y | Implant, expander/implant, LD/TRAM, DIEP, autol/implant | Self-designed questionnaire, 5-Likert scale/12 months | 72.3% general satisf., autol. group highest aesthetic satisf. (77.9%) |

BR, breast reconstruction; BRECON-31, Breast reconstruction satisfaction questionnaire; DIEP, Deep Inferior Epigastric Artery Perforator flap; DRS, Decision Regret Scale; EORTC BR23/QLQ C30 European Organisation for Research and Treatment of Cancer Breast Reconstruction/Quality of Life Questionnaire Physical function scale; FACT-B, Functional Assessment of Cancer Therapy – Breast; HADS, Hospital Anxiety and Depression Scale; LAM, Life After Mastectomy questionnaire; LD, latissimi dorsi; SF-12/36, 12-/36-item Short Form health survey; TD thoracodorsal; TE, tissue expansion; TILOOP, TiLoop® Bra mesh used for immediate breast reconstruction; (f)TRAM, (free) Transverse Rectus Abdominis Myocutaneous flap

# References

1. Ashraf, A.A., et al., *Patient involvement in the decision-making process improves satisfaction and quality of life in postmastectomy breast reconstruction.* J Surg Res, 2013. **184**(1): p. 665-70.

2. Augustinho, L.B.Z., et al., *Patient satisfaction with breast reconstruction using musculocutaneous flap from latissimus dorsi versus from rectus abdominis: A cross-sectional study.* Sao Paulo Medical Journal, 2018. **136**(6): p. 551-556.

3. Benditte-Klepetko, H.C., et al., *Analysis of patient satisfaction and donor-site morbidity after different types of breast reconstruction.* Scand J Surg, 2014. **103**(4): p. 249-55.

4. Caputo, G.G., et al., *Comparative study of patient outcomes between direct to implant and two-stage implant-based breast reconstruction after mastectomy.* J Plast Reconstr Aesthet Surg, 2021. **74**(10): p. 2573-2579.

5. Cordeiro, P., et al., *Extra-Full Projection Silicone Breast Implants: 2-Year Results from Two Prospective Studies.* Plast Reconstr Surg., 2015. **136**(4): p. 638-46.

6. Damen, T., et al., *High satisfaction rates in women after DIEP flap breast reconstruction.* J Plast Reconstr Aesthet Surg., 2010. **63**(1): p. 93-100.

7. Dauplat, J., et al., *Quality of life after mastectomy with or without immediate breast reconstruction.* British Journal of Surgery, 2017. **104**(9): p. 1197-1206.

8. de Blacam, C., et al., *Evaluation of clinical outcomes and aesthetic results after autologous fat grafting for contour deformities of the reconstructed breast.* Plast Reconstr Surg, 2011. **128**(5): p. 411e-418e.

9. Dean, N.R. and T. Crittenden, *A five year experience of measuring clinical effectiveness in a breast reconstruction service using the BREAST-Q patient reported outcomes measure: A cohort study.* J Plast Reconstr Aesthet Surg, 2016. **69**(11): p. 1469-1477.

10. Di Micco, R., et al., *Bilateral mammoplasty for cancer: Surgical, oncological and patient-reported outcomes.* European Journal of Surgical Oncology, 2017. **43**(1): p. 68-75.

11. Du, Z., et al., *Retrospective observational study of breast reconstruction with extended latissimus dorsi flap following skin-sparing mastectomy.* Medicine (Baltimore), 2018. **97**(31): p. e10936.

12. Fallbjork, U., et al., *Differences between women who have and have not undergone breast reconstruction after mastectomy due to breast cancer.* Acta Oncol, 2010. **49**(2): p. 174-9.

13. Gschwantler-Kaulich, D., et al., *Mesh versus acellular dermal matrix in immediate implant-based breast reconstruction - A prospective randomized trial.* Eur J Surg Oncol, 2016. **42**(5): p. 665-71.

14. Heneghan, H.M., et al., *Quality of life after immediate breast reconstruction and skin-sparing mastectomy - a comparison with patients undergoing breast conserving surgery.* Eur J Surg Oncol, 2011. **37**(11): p. 937-43.

15. Ho, P.J., et al., *Determinants of satisfaction with cosmetic outcome in breast cancer survivors: A cross-sectional study.* PLoS ONE, 2018. **13**(2).

16. Howes, B.H.L., et al., *Quality of life following total mastectomy with and without reconstruction versus breast-conserving surgery for breast cancer: A case-controlled cohort study.* Journal of Plastic, Reconstructive and Aesthetic Surgery, 2016. **69**(9): p. 1184-1191.

17. Hunsinger, V., et al., *Long-Term Follow-Up of Quality of Life following DIEP Flap Breast Reconstruction.* Plast Reconstr Surg, 2016. **137**(5): p. 1361-1371.

18. Juhl, A.A., et al., *Unilateral breast reconstruction after mastectomy - patient satisfaction, aesthetic outcome and quality of life.* Acta Oncol, 2017. **56**(2): p. 225-231.

19. Kelsall, J.E., et al., *Comparing oncoplastic breast conserving surgery with mastectomy and immediate breast reconstruction: Case-matched patient reported outcomes.* J Plast Reconstr Aesthet Surg, 2017. **70**(10): p. 1377-1385.

20. Liu, H.H., et al., *Postmastectomy breast reconstruction combined with contralateral breast augmentation for taiwanese women with small breasts.* Annals of Plastic Surgery, 2017. **78**(3): p. S102-S107.

21. Liu, Q., et al., *Immediate breast reconstruction using latissimus dorsi muscular flap: A retrospective study of Chinese patients with breast cancer.* Medicine (Baltimore), 2021. **100**(24): p. e26175.

22. Mericli, A.F., et al., *The Latissimus Dorsi Myocutaneous Flap Is a Safe and Effective Method of Partial Breast Reconstruction in the Setting of Breast-Conserving Therapy.* Plast Reconstr Surg, 2019. **143**(5): p. 927e-935e.

23. Momoh, A.O., et al., *Analysis of complications and patient satisfaction in pedicled transverse rectus abdominis myocutaneous and deep inferior epigastric perforator flap breast reconstruction.* Ann Plast Surg, 2012. **69**(1): p. 19-23.

24. Nelson, J.A., et al., *Long-term Patient-reported Outcomes Following Postmastectomy Breast Reconstruction: An 8-year Examination of 3268 Patients.* Ann Surg, 2019. **270**(3): p. 473-483.

25. Qin, Q., et al., *Postoperative outcomes of breast reconstruction after mastectomy: A retrospective study.* Medicine (Baltimore), 2018. **97**(5): p. e9766.

26. Rindom, M.B., et al., *Good health-related quality-of-life and high patient-reported satisfaction after delayed breast reconstruction with pedicled flaps from the back.* J Plast Reconstr Aesthet Surg, 2021. **74**(8): p. 1752-1757.

27. Roje, Z., et al., *Breast reconstruction after mastectomy.* Collegium Antropologicum, 2010. **34**(SUPPL. 1): p. 113-123.

28. Santosa, K.B., et al., *Effect of Patient Age on Outcomes in Breast Reconstruction: Results from a Multicenter Prospective Study.* J Am Coll Surg, 2016. **223**(6): p. 745-754.

29. Santosa, K.B., et al., *Long-term Patient-Reported Outcomes in Postmastectomy Breast Reconstruction.* JAMA Surg, 2018. **153**(10): p. 891-899.

30. Semmler, V., *Vergleich der Lebensqualität nach Latissimus Dorsi Lappen (LDF) und Unterbauchlappen (DIEP) zur Mammarekonstruktion*, in *Abteilung für Plastische und Rekonstruktive Chirurgie*. 2012, Ruhr-Universität Bochum.

31. Sgarzani, R., et al., *Patient Satisfaction and Quality of Life in DIEAP Flap versus Implant Breast Reconstruction.* Surg Res Pract, 2015. **2015**: p. 405163.

32. Spector, D.J., et al., *Women's recovery experiences after breast cancer reconstruction surgery.* J Psychosoc Oncol, 2011. **29**(6): p. 664-76.

33. Szutowicz-Wydra, B., et al., *Same Quality of life for Polish Breast cancer patients treated with mastectomy and Breast reconstruction or breast-conserving therapy.* Polski Przeglad Chirurgiczny/ Polish Journal of Surgery, 2016. **88**(5): p. 264-269.

34. Tallroth, L., P. Velander, and S. Klasson, *A short-term comparison of expander prosthesis and DIEP flap in breast reconstructions: A prospective randomized study.* J Plast Reconstr Aesthet Surg, 2021. **74**(6): p. 1193-1202.

35. Temple-Oberle, C., et al., *Shared decision-making: applying a person-centered approach to tailored breast reconstruction information provides high satisfaction across a variety of breast reconstruction options.* J Surg Oncol, 2014. **110**(7): p. 796-800.

36. Wang, X., et al., *Nipple-areola sparing mastectomy followed by immediate breast reconstruction in 126 patients.* Journal of Cancer Therapy, 2012. **3**(5): p. 831-835.

37. Wattoo, G., et al., *Long-term outcomes of latissimus dorsi flap breast reconstructions: A single-centre observational cohort study with up to 12 years of follow up.* J Plast Reconstr Aesthet Surg, 2021. **74**(9): p. 2202-2209.

38. Xu, F., et al., *Multi-center investigation of breast reconstruction after mastectomy from Chinese Society of Breast Surgery: A survey based on 31 tertiary hospitals (CSBrS-004).* Chin J Cancer Res, 2021. **33**(1): p. 33-41.
